# *Tfl* deletion induces extraordinary Cxcl13 secretion and cachexia in VavP-*Bcl2* transgenic mice

**DOI:** 10.3389/fimmu.2023.1197112

**Published:** 2023-05-26

**Authors:** Kentaro Minagawa, Kanako Wakahashi, Chie Fukui, Yuko Kawano, Hiroki Kawano, Tomohide Suzuki, Shinichi Ishii, Akiko Sada, Shinichiro Nishikawa, Noboru Asada, Yoshio Katayama, Toshimitsu Matsui

**Affiliations:** ^1^ Hematology, Department of Medicine, Kobe University Graduate School of Medicine, Kobe, Japan; ^2^ Hematology & Oncology Division, Penn State College of Medicine, Hershey, PA, United States; ^3^ Department of Hematology, Nishiwaki Municipal Hospital, Nishiwaki, Japan

**Keywords:** Regnase-4, MCPIP-4, RNA regulation, Bcl-2, cachexia

## Abstract

Statement of significance: Loss of *TFL*, found in several types of lymphoma, induces excessive CXCL13 secretion through RNA dysregulation contributing to body weight loss and early death in lymphoma model mice. Follicular lymphoma (FL) is associated with overexpressed BCL-2 and other genetic aberrations, including 6q-. We identified a novel gene on 6q25, “*Transformed follicular lymphoma* (*TFL*),” from a transformed FL. TFL regulates several cytokines *via* mRNA degradation, which has been suggested to underlie resolving inflammation. Fluorescence *in situ* hybridization revealed a deletion of *TFL* occurred in 13.6% of various B-cell lymphoma samples. We developed VavP-*bcl2* transgenic, TFL deficit mice (*Bcl2*-Tg/*Tfl* ^-/-^) to seek how TFL affects disease progression in this lymphoma model. While *Bcl2*-Tg mice developed lymphadenopathy and died around 50 weeks, *Bcl2*-Tg/*Tfl* ^-/-^ mice lost body weight around 30 weeks and died about 20 weeks earlier than *Bcl2*-Tg mice. Furthermore, we found a unique B220^-^IgM^+^ cell population in the bone marrow of *Bcl2*-Tg mice. cDNA array in this population revealed that Cxcl13 mRNA in *Bcl2*-Tg/*Tfl* ^-/-^ mice expressed significantly higher than *Bcl2*-Tg mice. In addition, bone marrow extracellular fluid and serum showed an extremely high Cxcl13 concentration in *Bcl2*-Tg/*Tfl* ^-/-^ mice. Among bone marrow cells, the B220^-^IgM^+^ fraction was the main producer of Cxcl13 in culture. A reporter assay demonstrated TFL regulates CXCL-13 *via* induction of 3’UTR mRNA degradation in B lineage cells. These data suggest Tfl regulates Cxcl13 in B220^-^IgM^+^ cells in the bone marrow, and a very high concentration of serum Cxcl13 arising from these cells may contribute to early death in lymphoma-bearing mice. Since several reports have suggested the association of CXCL13 expression with lymphoma, these findings provide new insights into cytokine regulation *via* TFL in lymphoma.

## Introduction

Follicular lymphoma (FL) is amongst the most common B cell lymphoma and often overexpress BCL-2 caused by t(14;18)(q32;q21) translocation. Some FL transforms into diffuse large B cell lymphomas (DLBCL) during long-time follow-up. In addition to t(14;18), several genetic aberrations have been reported, including losses of 1p and 6q (1). These segmental deletions have been linked to tumorigenesis (2, 3) and poor prognosis (4). On the other hand, microenvironmental interactions are critical for lymphoma pathogenesis. Follicular dendritic cells in the germinal center express B cell activating factor (BAFF) and secrete CXCL13. BAFF and CXCL13 activate FL through binding BAFF receptor or CXCR5 on FL cells, respectively. CXCL13 also activates follicular helper T-cells that express ICOS, CD40L, and TCR and support FL progression (1). CXCL13 secretion was seen in FL cells (5), and higher serum CXCL13 levels for more than 3 years have been associated with an increased risk of development of HIV-associated non-Hodgkin lymphoma (6). A recent survey showed that higher serum CXCL13 level predicts future DLBCL (7). These results suggest that inflammatory signaling may change lymphoma development and prognosis.

We identified a novel gene called ***T*** *ransformed* ***F*** *ollicular* ***L*** *ymphoma (TFL)* on 6q25 from a patient with transformed follicular lymphoma (8–10). TFL contains a unique RNase NYN/PIN-like motif, which is considered to be crucial for its biological effect (11). TFL regulates several cytokines, including IL-2, IL-6, and IL-17a, *via* 3’UTR binding and mRNA degradation and affects the resolution of autoimmune encephalopathy (12). A recent report showed that 12% of DLBCL have a loss of function of *ZC3H12A* (also known as MCPIP-1 and Regnase-1), a TFL family member (13). We hypothesized that loss of TFL function might cause cytokine dysregulation in lymphoma, worsening prognosis. This study aimed to determine the frequency of TFL loss in lymphoid malignancies and further evaluate cytokine dysregulation by Tfl on disease outcomes using *Bcl-2* transgenic (Tg) mice as an FL model.

## Materials and methods

### FISH analysis

Patients’ leftover cells fixed in Carnoy’s solution were used for FISH analysis under IRB approval (#605). Patients diagnosed with lymphoma were asked to participate in the study. The fixed samples were stored at -20°C until use. To perform DNA-FISH, cells were spotted onto glass slides and let dry. After pepsin treatment (2% pepsin in 0.01N HCl, 1 h at room temperature), cells were washed in PBS and dehydrated through an ethanol gradient (70%, 90%, and 100% for 10 min each). Samples were heated at 80°C for 3 min to separate DNA strands, then 1.25–12.5 ng/μL of probes in hybridization buffer (4xSSC, 0.5 mM EDTA, 10% dextran sulfate, 25% deionized-formamide in deionized H_2_O) were applied and incubated at room temperature for 2 h before imaging. Locus Specific Identifier (LSI) probe for TFL was prepared from clone RP1-281H8 on chromosome 6q25.1-25.3 (BACPAC Resources Center) with QIAGEN Large-Construct Kit (Qiagen, Gaithersburg, MD) and green fluorescein-labeled Vysis CEP 6 (D6Z1) (Abbott, Lake Bluff, IL) was used as centromere probe (CEP). LSI probe was labeled with orange dUTP with nick translational kit (Abbott). At least 100 cells were evaluated, and when 5% or more of the evaluated cells were CEP>LSI, these samples were defined as loss of *TFL* locus based on the background in the normal samples. On the contrary, when 5% or more of evaluated cells were CEP<LSI, these samples were defined as a gain of *TFL* locus. Otherwise, samples were determined as having no *TFL* deletion (CEP=LSI).

Bone marrow, lymph node, and tissue samples of patients diagnosed with hematological malignancies were collected for the purpose of diagnosis. The leftover samples were used for FISH and RNA blot analysis with written informed consent or an opt-out approach under institutional review board approval at Kobe University (approval #605).

### RNA blot analysis

RNA blots were hybridized with ^32^P-labeled human *TFL* cDNA probes. A 963-bp human TFL probe, which includes the complete open reading frame of TFL2 (10), was amplified using a forward primer 5’-ATGGAGCACCCCAGCAAGATGGAATTC-3’, and a reverse primer, 5’-CTACCCACCATAAGGACAATGCTGC-3’. The image was measured with the ImageJ software (ImageJ, RRID: SCR_003070). Ethidium bromide (EtBr) was used as a loading control. Each region of interest for RNA blot and EtBr image was gated, and the pixel density was captured. The relative quantification value is determined by the ratio of the net band to net loading control.

### Mice

Mice were under the husbandry care of the Institute for Experimental Animals, Kobe University Graduate School of Medicine. *Tfl* ^-/-^ mice generated by gene targeting (Minagawa et al.) were back-crossed for more than twelve generations into a C57BL/6 background. VavP-*Bcl2*-Tg mice were kindly provided by Dr. Cory and Dr. Bouillet (The Walter and Eliza Hall Institute of Medical Research, Department of Medical Biology, University of Melbourne). Wild-type C57BL/6 mice were purchased from CLEA Japan (Chiba, Japan). Animals were maintained under specific pathogen-free conditions and on 12 h light/12 h darkness cycle. Both female and male mice were used in these studies otherwise specified. The Animal Care and Use Committee of Kobe University approved all animal studies.

### Immunohistochemistry

Paraffin-embedded sections were used for the immunohistochemical staining of Peanut Agglutinin (PNA), Ki-67, and NFκB1. After inhibiting endogenous peroxidase activity with methanol-containing 0.3% hydrogen peroxidase for 30 min, antigen retrieval was done with citrate buffer (pH 6.0). Sections were blocked with 5% goat serum (Jackson Immunoreseach Labs, West Grove, PA) and 5% FBS (Thermo Fisher Scientific, Waltham, MA)/PBS for 1 h. Primary biotinylated PNA (Vector Laboratories, Burlingame, CA), or anti-Ki-67 or NFκB1 antibody (Abcam, Cambridge, MA) was used at optimized dilutions and detected by the avidin-biotin-horseradish peroxidase complex (ABC) system (Vector Laboratories) using horseradish peroxidase (HRP), or HRP polymer for mouse tissue, Histostar™ (MBL, Nagoya, Japan). Sections were counterstained with hematoxylin. Proportion of positive cells were calculated with the ImageJ software.

### Flow cytometry and cell sorting

The reagents for flow cytometry were from BD Biosciences (San Jose, CA). Cells were suspended in PBS/0.5% BSA/2 mM EDTA. Cell analyses were performed on BD FACScan and Accuri flow cytometer with BD CellQuest and FlowJo software (FlowJo, RRID: SCR_008520, BD Biosciences). Live-dead cell and singlet gating was performed during the analysis and cell sorting. For sorting of bone marrow cells (B220^+^, B220^-^IgM^-^, or B220^-^IgM^+^), bone marrow cells were harvested by flushing the femur aseptically in RPMI 1640 (Sigma-Aldrich, St. Louis, MO) and single-cell suspension was obtained by gentle aspiration several times. Cells were washed with PBS/0.1% BSA/2 mM EDTA and stained with FITC-anti-IgM (BD Pharmingen, San Diego, CA) and PE-anti-B220 (BD Pharmingen) antibodies. Cell analysis and sorting were performed on a MoFlo XDP flow cytometer with Summit software (Beckman Coulter, Brea, CA).

### Cell culture

Bone marrow cells were obtained as described above. Splenocytes were harvested by gentle aspiration with RPMI 1640. Soon after bone marrow cells and Splenocytes were harvested, and fractionated bone marrow cells (B220^+^, B220^-^IgM^-^, or B220^-^IgM^+^ cells) were sorted, they were cultured at a concentration of 10^6^ cells/well in 500μL RPMI 1640 supplemented with 10% FBS in a non-coated 24-well dish at 37°C, 5% CO^2^. The cell culture supernatant was harvested for Cxcl13 ELISA at 96 h.

### cDNA array

We compared RNAs of bone marrow B220^-^IgM^+^ cells from VavP-*Bcl2*Tg and Vavp-*Bcl2*Tg/*Tfl* ^-/-^ in the two independent experiments. We used a cDNA array chip with SurePrint G3 Mouse GE microarray kit 8x60k (G4852A, database: mm9:NCBI37:Jul2007, Agilent Technologies, Santa Clara, CA). The chip was hybridized with labeled cDNA probes prepared by reverse transcription from 100 ng polyA mRNA using the respective protocol from Agilent Technologies. Probes used for hybridization were labeled with Cy3 dye. Overnight incubation was followed by stringent washing as recommended by the manufacturer. The hybridization signals were scanned and evaluated with a scanner (G2539A) and Scan Control software (version A.8.5.1) and digitalized with Feature Extraction software (Agilent Technologies, version 10.10.1.1). The obtained data were analyzed with GeneSpring GX (version 12.6.1, Agilent Technologies). These procedures were outsourced to Oncomics (Lui-dong, Korea).

### RNA extraction and Real-time PCR

Samples were mixed with 1.5 ml TRIzol (Life Technologies, Carlsbad, CA) and stored at -80 ˚C. RNA extraction and quantitative RT-PCR (qPCR) were performed as described previously (14). Briefly, qPCR was performed with gene-specific primers with SYBR Green dye (ThermoFisher Scientific, Waltham, MA). The sequence of each primer is available upon request. Each gene expression was normalized with the value of beta-actin. For the cDNA array, RNAs were purified with an RNeasy kit (Qiagen). The primers used are available upon request.

### Cxcl13 ELISA assay

Quantification for Cxcl13 in bone marrow extra fluid, plasma, and supernatant of culture medium was done using a mouse Cxcl13 ELISA Kit (R&D systems, Minneapolis, MN).

### Luciferase reporter assay

The luciferase assay was performed as described previously (14). Briefly, HeLa cells (CLS Cat# 300194/p772_HeLa, RRID: CVCL_0030) were transfected with psiCheck2 plasmids (Promega, Madison, WI) with or without 3’UTR of CXCL13 or IL-2. Cells were co-transfected either with the control or TFL expression plasmid. For suspension cells, Amaxa nucleofector (Lonza, Basel, Swiss) was used for the transfection of those vectors according to the manufacturer’s developed protocols. Luciferase activity was determined using the Dual-Luciferase reporter assay system (Promega, Madison, WI). The expression value of Renilla Luciferase (vector construct containing 3’UTR) was normalized by Firefly Luciferase (internal control). Experiments were triplicated, and the median value was chosen for the data. Obtained data with the TFL vector were further normalized by those with the control vector (no TFL expression). We confirmed >10,000 relative light units (RLU) in Renilla Luciferase and >1,000 RLU in Firefly Luciferase activity to ensure the transfection efficacy in each cell line. Each experiment was performed at least 3 times to show standard error and perform statistical analysis.

### Statistics

All data are presented as the means ± SEM. Kaplan-Meier with log-rank test was used to estimate survival curves and for statistical comparison of the two groups. The student’s t-test was applied when comparing two independent groups. All the statistical analyses were performed using GraphPad Prism software (GraphPad Prism, RRID: SCR_002798, GraphPad, San Diego, CA).

## Results

### Loss of *TFL* locus in mature B cell neoplasms

**Figure 1:**
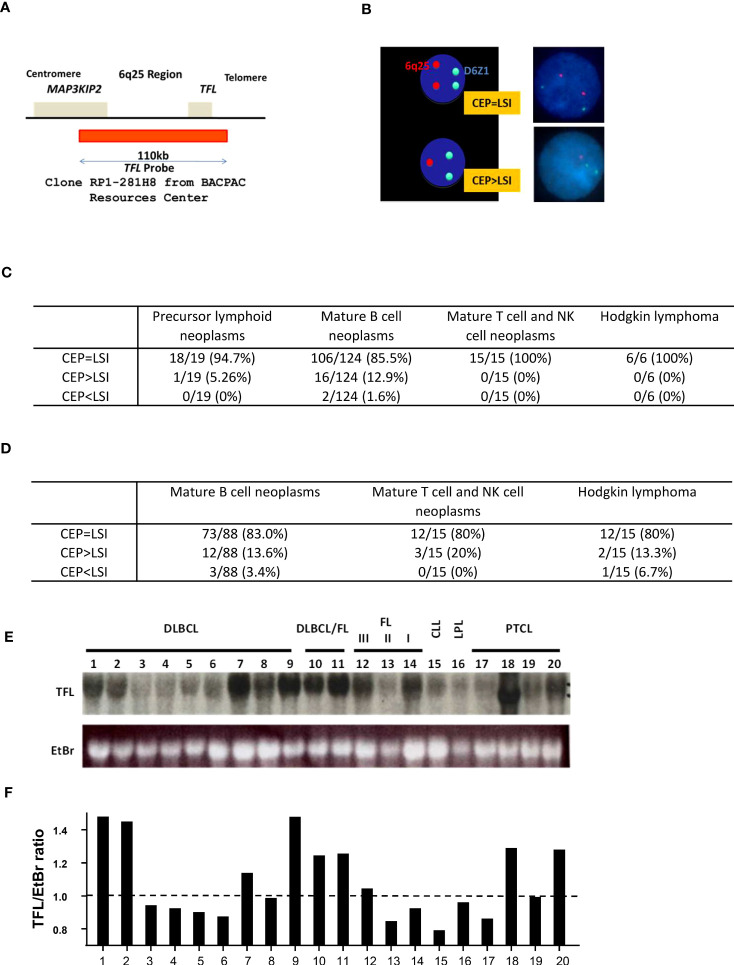
Loss of TFL locus in mature B cell neoplasms. **(A)** TFL DNA probe used for FISH analysis was derived from RP1-281H8 (BACPAC Resources Center). It covered 110kbp of the 6q25 region, which contains part of *MAP3KIP2* and *TFL*. **(B)** D6Z1 was used as a centromere probe (CEP). When the number of CEP is equal to that of a local specific identifier (LSI; here, TFL probe), TFL is not considered to be deleted. When the number of LSI is less than that of CEP, TFL is considered to be deleted. Typical normal staining is shown. **(C)** The results of FISH analysis for bone marrow samples. **(D)** The results of FISH analysis for lymph nodes (LN) or tissue samples. **(E)** RNA blot analysis for various lymphoma tissue samples. TFL: Transformed follicular lymphoma, EtBr: Ethidium bromide, DLBCL: Diffuse large B cell lymphoma, DLBCL/FL: Follicular lymphoma transformed into DLBCL, FL III/II/I: Follicular lymphoma grade III/II/I, CLL: Chronic lymphocytic leukemia, LPL: Lymphoplasmacytic lymphoma, PTCL: Peripheral T cell lymphoma. **(F)** Quantification of RNA blot analysis is shown in **(E)**. The image was measured and normalized by the value of EtBr.

First, we sought clinical relevance between the loss of TFL and the development of lymphoid neoplasms. We made a fluorescence *in situ* hybridization (FISH) probe for *TFL* locus detection, covering 110 kbp around TFL located lesion, 6q25 (**Figures 1A, B**), and tested FISH analysis for bone marrow and lymph node or tissue samples from lymphoid malignancy patients (IRB#605). In 164 bone marrow samples, 124 samples were diagnosed as mature B cell neoplasm, of which 12.9% of patients showed TFL locus deletion (16 in 124 patients; **Figure 1C**, **Supplemental Table 1**). For the lymph node or tissue samples, 13.6% of patients diagnosed with mature B cell neoplasm were detected as TFL deletion (12 in 88 patients; **Figure 1D**, **Supplemental Table 2**). TFL locus deletion was also seen in other types of lymphoma, such as mature T and NK cell neoplasm (3 in 15 patients) and Hodgkin lymphoma (2 in 15 patients). In FL, 2 patients with TFL loss in the bone marrow samples were Grade 2, and 6 patients with TFL loss in tissue samples were 5 with Grade 2 and 1 with Grade 3, while no TFL deletion was found in those with Grade 1. To confirm the loss of TFL expression, we performed an RNA blot analysis for several lymphoma/leukemia patients. This experiment showed several patients with mature B lymphoid malignancy decreased TFL expression (Patients #3-6 and 13-16; **Figures 1E, F**). These data indicate the loss of TFL expression has occurred in mature B cell neoplasms frequently. For FL and DLBCL, TFL expression increased in higher grades of FL and transformed FL (FL/DLBCL) (Patients #10-12), while decreased TFL was seen more in DLBCL (Patients # 3-6).

### *Tfl* deficiency does not affect tumorigenesis but shortens the survival of *Bcl2*-Tg mice

**Figure 2:**
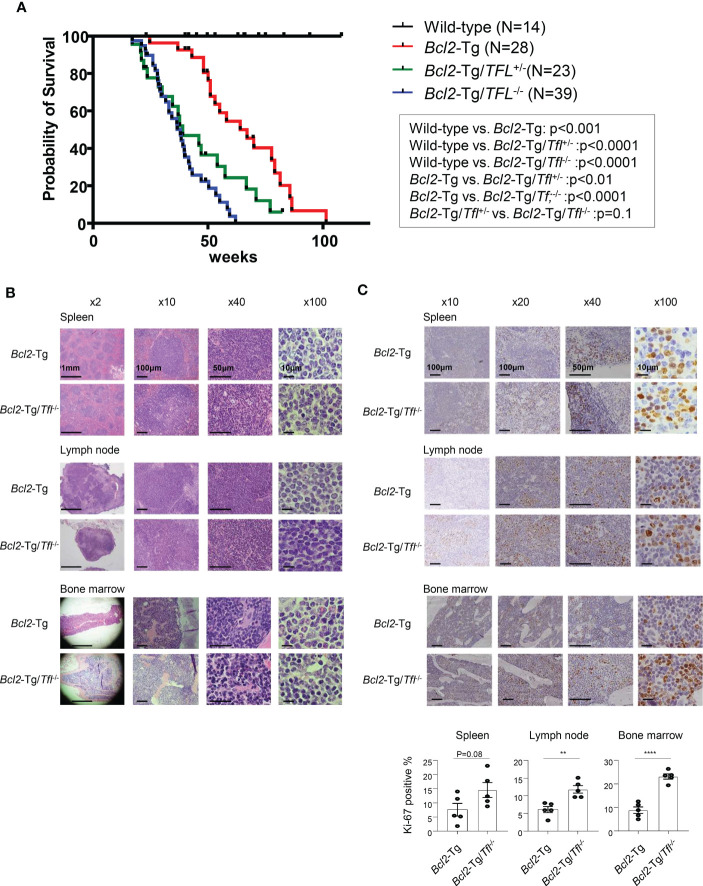
Survival of *Bcl2*-Tg/*Tfl* ^-/-^ mice. **(A)** A Kaplan-Meier plot for wild type (N=14), *Bcl2*-Tg (N=28), *Bcl2*-Tg/*Tfl* ^+/-^ (N=23) and *Bcl2*-Tg/*Tfl* ^-/-^ mice (N=39). **(B)** Histological analysis of *Bcl2*-Tg and *Bcl2*-Tg/*Tfl* ^-/-^ mice. Hematoxylin and eosin (HE) staining was shown for the spleen (upper), lymph nodes (middle), or bone marrow (lower panel). **(C)** Ki-67 immunostaining was shown for the spleen (upper), lymph nodes (middle), or bone marrow (lower panel). For each section, at least three pairs of mice were analyzed. The representative pictures were shown. The percentage of positive cells was shown. Positive cells were counted for 5 independent areas in each organ. The p-value is shown as **<0.01 and ****<0.0001.

*Tfl* ^-/-^ mouse was developed in our lab previously (12). Their median survival was 119 week (range 82-144 weeks), comparable to wild-type C57BL/6 mice (the median survival: 108-116 weeks). To determine the effect of tumorigenesis caused by loss of TFL in matured B cell neoplasms, we utilized vavP-*Bcl2* transgenic (*Bcl2*-Tg) mice as FL-bearing mice (15). For this aim, we developed VavP-*Bcl2*-Tg/TFL deficit (*Bcl2*-Tg/*Tfl* ^-/-^) mice and observed their survival periods. *Bcl2*-Tg mice developed lymphadenopathy and splenomegaly and died around 60 weeks (median survival of 64 weeks, **Figure 2A**), which is significantly shorter than wild-type mice suggesting follicular lymphoma development as reported. Surprisingly, *Bcl2*-Tg/*Tfl* ^-/-^ mice died around 20 weeks earlier than *Bcl2*-Tg mice (median survival of 38 weeks, p<0.0001). These phenomena occurred in *Bcl2*-Tg/*Tfl* ^+/-^ mice and both female and male mice (**Figure 2A** and **Supplemental Figure 1**). While no sex biases were seen in *Bcl2*-Tg and *Bcl2*-Tg/*Tfl* ^-/-^ mice, female *Bcl2*-Tg/*Tfl* ^+/-^ mice died earlier than male mice. *Bcl2*-Tg/*Tfl* ^-/-^ mice showed similar lymphadenopathy, and the size of splenomegaly was not different from *Bcl2*-Tg (data not shown). We performed several necropsies, but Bcl2-Tg/*Tfl* ^-/-^ mice showed no other malignant tumor except for lymphadenopathy (data not shown). Morphological and histological findings in the spleen, lymph nodes, and bone marrow between those two strains showed no significant difference (**Figure 2B**). No significant morphological transformation of lymphocytes was seen in Bcl2-Tg/*Tfl* ^-/-^ mice. Lymphocyte infiltration patterns in the kidney, liver, and lung were also similar between the two groups (**Supplemental Figure 2**). On the other hand, increased Ki-67 positive cells were noted in *Bcl2*-Tg/*TFL* ^-/-^ mice in the spleen, lymph nodes, and bone marrow compared with *Bcl2*-Tg mice (**Figure 2C**).

### *Tfl* deficiency induces body weight loss in *Bcl2*-Tg mice

**Figure 3:**
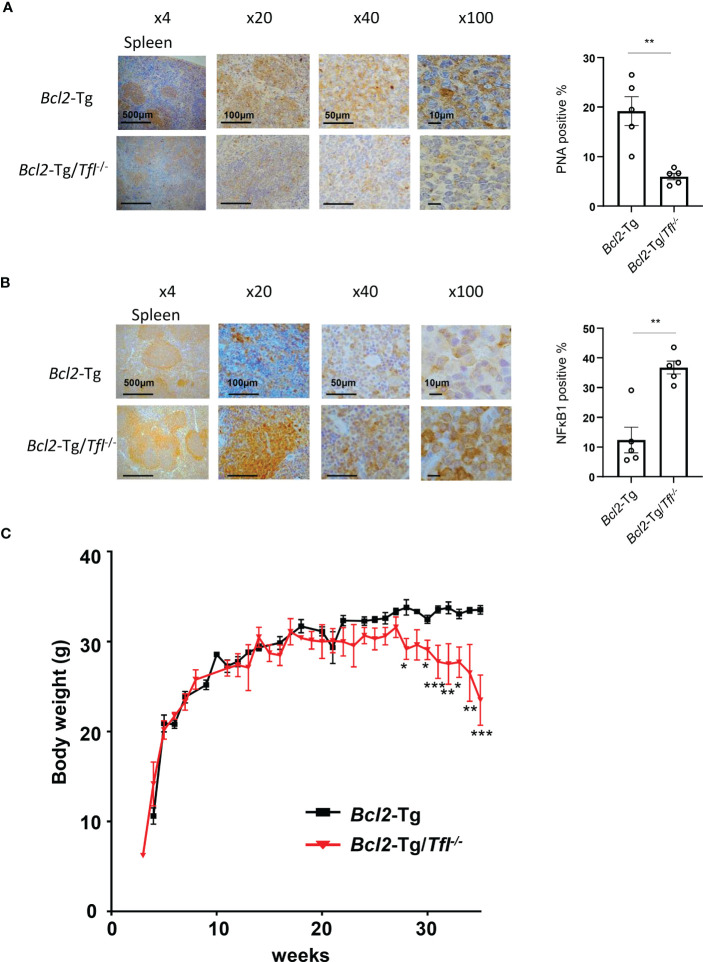
*Bcl2*-Tg/*Tfl* ^-/-^ mice decrease their body weight. Peanut agglutinin lectin (PNA) immunohistochemical staining **(A)** and NFκB1 immunostaining **(B)** in the spleen of *Bcl2*-Tg/*Tfl* ^+/+^ and *Bcl2*-Tg/*Tfl* ^-/-^ mice were shown (Left). The percentage of positive cells was shown (Right). Positive cells were counted for 5 independent areas. **(C)** Body weight change of male *Bcl2*-Tg (N=17), and *Bcl2*-Tg/*Tfl* ^-/-^ mice (N=18). The *p*-value was shown as *<0.05, **<0.01, and ***<0.001.

Next, we performed peanut agglutinin (PNA) staining of spleen sections to visualize germinal center B cell proliferation. *Bcl2*-Tg/*Tfl* ^-/-^ mice showed reduced germinal center B cell accumulation in the follicles compared with *Bcl2*-Tg (**Figure 3A**). MCPIP-1, a TFL family member, has been shown to demonstrate deubiquitination affecting the NFκB pathway (16), and TFL is also suggested to regulate the NFκB pathway (17). NFκB1 in the spleen of *Bcl2*-Tg and *Bcl2*-Tg/*Tfl* ^-/-^ mice expressed mainly interfollicular area (**Figure 3B**), indicating highly activated intrafollicular B cells in this area (18). Interestingly, increased NFκB1 positive cells were observed in *Bcl2*-Tg/*Tfl* ^-/-^ mice spleen compared with *Bcl2*-Tg mice. Complete blood counts in peripheral blood showed no significant differences between both groups except platelet counts (p<0.05, **Supplemental Figure 3A**). When stratified the results into blood counts at 20-30 weeks and 35-45 weeks, MCV and MCH were significantly decreased (p<0.05), and platelet count tended to decrease (p=0.052) in *Bcl2-*Tg/*Tfl* ^-/-^ mice at 35-45 weeks (**Supplemental Figure 3B**). Interestingly, the body weight of *Bcl2-*Tg/*Tfl* ^-/-^ mice began to decrease at week 25 and nadired at week 35 compared with *Bcl2*-Tg (**Figure 3C**), suggesting that this body weight loss may contribute to shorter survival.

### Extraordinary Cxcl13 secretion in *Bcl2*-Tg/*Tfl^-/-^* bone marrow

**Figure 4:**
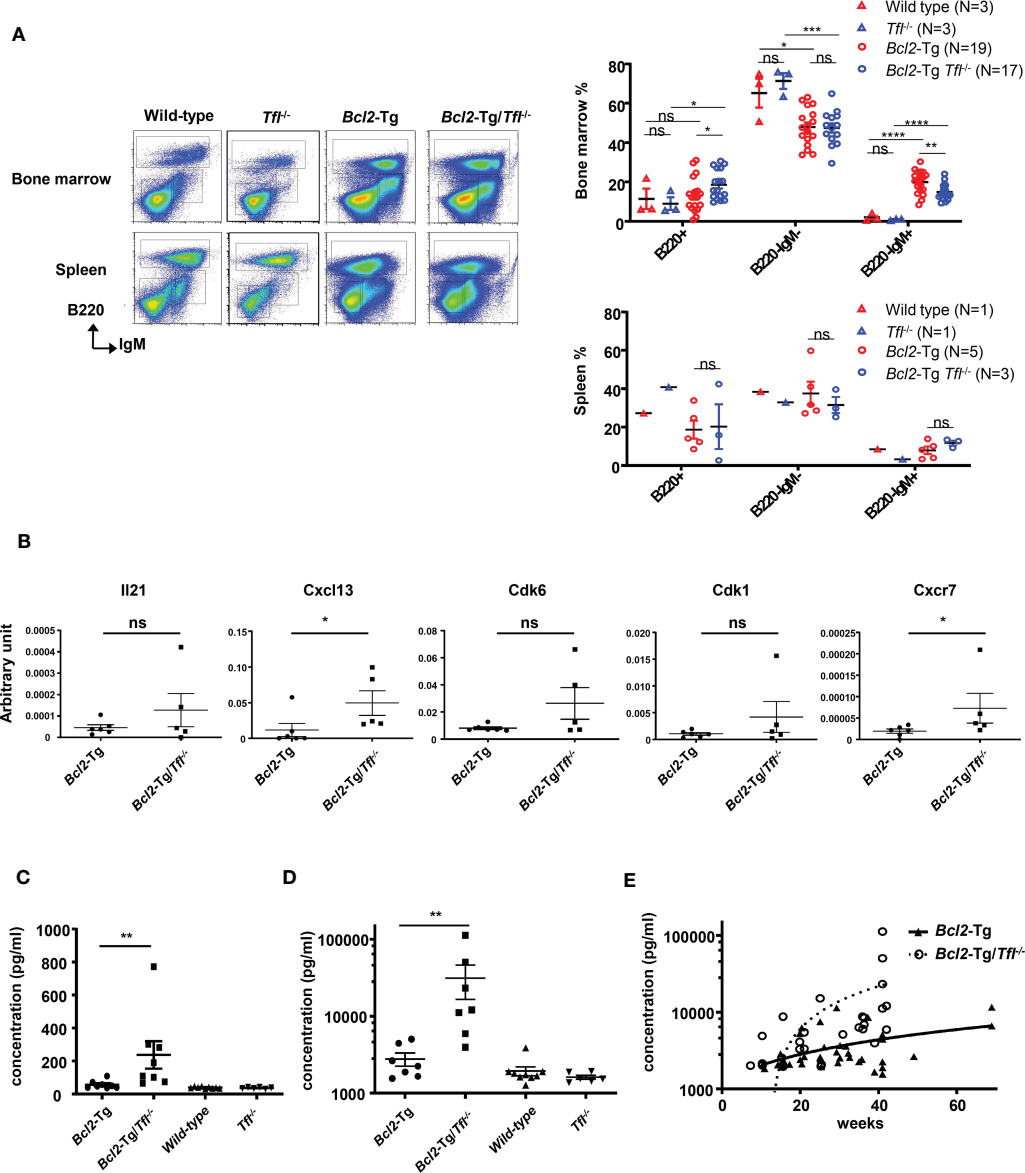
Increased Cxcl13 secretion in *Bcl2*-Tg/*Tfl^-/-^* mice. **(A)** Representative dot plots (Left panel) of cells from bone marrow or spleen of wild type, *Tfl* ^-/-^, *Bcl2*-Tg, or *Bcl2-*Tg/*Tfl* ^-/-^ mice. Each B220^+^, B220^-^IgM^-^, and B220^-^IgM^+^ fraction is shown in rectangles. The percentage of each fraction in wild-type, *Tfl* ^-/-^, *Bcl2*-Tg, or *Bcl2-*Tg/*Tfl* ^-/-^ mice are shown for bone marrow (Right upper panel) and spleen (Right lower panel). **(B)** Real-time PCR analysis for Il-21, Cxcl13, Cdk6, Cdk1, and Cxcr7 in the B220^-^IgM^+^ bone marrow cells between *Bcl2*-Tg (N=6) and *Bcl2-*Tg/*Tfl* ^-/-^ (N=5) mice. *P* values are shown in the figure. Cxcl13 measurement of bone marrow extra fluid **(C)** or plasma **(D)** at 40-44 weeks from *Bcl2*-Tg (N=7-8) and *Bcl2-*Tg/*Tfl* ^-/-^ (N=7-8) mice. Data from wild-type (N=9) and *Tfl* ^-/-^ (N=6) mice are shown as references. The course of Cxcl13 measurement of plasma **(E)** from *Bcl2*-Tg (N=38) and *Bcl2*-Tg/*Tfl* ^-/-^ (N=28) mice. The *p*-value is shown as ns (not significant), *<0.05, **<0.01, ***<0.001, and ****<0.0001.

To clarify the causes of earlier death in *Bcl2*-Tg/*TFL* ^-/-^ mice, we carefully examined their bone marrow and splenocyte phenotypes. We found a prominent B220^-^IgM^+^ population in the bone marrow of *Bcl2*-Tg mice, which was rare in *Tfl* ^-/-^ and wild-type mice (**Figure 4A**: Wild type 2.0 ± 1.9%, *Tfl* ^-/-^ 0.9 ± 0.4%, *Bcl-2*-Tg 20.0% ± 5.8%, *Bcl2*-Tg/*Tfl* ^-/-^ 14.9% ± 4.1%). While this B220^-^IgM^+^ proportion of *Bcl2*-Tg/*Tfl* ^-/-^ was decreased compared with *Bcl2*-Tg (p<0.01), total cell counts of this population were increased (mean cell count of the wild type was 2.1 x 10^5^, *Tfl* ^-/-^ 2.1 x 10^5^, *Bcl-2*-Tg 27.6 x 10^5^, and *Bcl2*-Tg/*Tfl* ^-/-^ 31.7 x 10^5^, per femur respectively, N=2). This population was positive for CD25, CD11b, and Igκ and partially positive for CD4, Gr-1, and CXCR4 (**Supplemental Figure 4**). We also confirmed F4/80 expression (94.7%) in Bcl2-Tg mice (data not shown). This population contains a distinctive small fraction of T-cells (CD3+), erythroid (Ter119+), and myeloid progenitor cells (Gr-1+). No significant phenotypic difference was seen in the B220^-^IgM^+^ fraction between *Bcl-2*-Tg, and *Bcl2*-Tg/*Tfl* ^-/-^ bone marrow. Although a B220^-^IgM^+^ population was found in both wild type and *Bcl2*-Tg spleen, it was less prominent (Wild type 8.4%, *Tfl* ^-/-^ 3.3%, *Bcl-2*-Tg 7.9% ± 4.4%, *Bcl2*-Tg/*Tfl* ^-/-^ 11.8% ± 2.3%). Therefore, we thought this unique population could affect lymphomagenesis in *Bcl2*-Tg mice. We speculated that the function of this unusual cell population could be altered by TFL deficiency, resulting in shorter survival in *Bcl2*-Tg/*Tfl^-/-^* mice. To elucidate the functional changes in these populations, we performed a cDNA expression array using B220^-^IgM^+^ sorted bone marrow cells of both *Bcl2*-Tg and *Bcl2*-Tg/*Tfl* ^-/-^ mice (**Supplemental Files 1, 2**). In these cells in *Bcl2*-Tg/*Tfl* ^-/-^ mice, 182 genes were upregulated at least two-fold relative to *Bcl2*-Tg mice (**Supplemental File 3**). Among them, we selected Il-21, Cxcl13, Cdk6, Cdk1, and Cxcr7, all cytokine, cell cycle, or cancer-related genes. Their mRNA expression was confirmed by real-time quantitative PCR. Among these upregulated genes, Cxcl13 and Cxcr7 mRNA expression in *Bcl2*-Tg/*Tfl* ^-/-^ mice was significantly higher than *Bcl2*-Tg mice, although the expressions varied in each *Bcl2*-Tg/*Tfl* ^-/-^ mice (**Figure 4B**). To further confirm Cxcl13 overexpression, we measured Cxcl13 concentration in bone marrow extracellular fluid (BMEF) and plasma samples in both strains at week 40-44. We found a significant increase in Cxcl13 concentration in *Bcl2*-Tg/*Tfl* ^-/-^ mice compared to *Bcl2*-Tg mice in BMEF (**Figure 4C**). Plasma Cxcl13 levels in *Bcl2*-Tg/*Tfl* ^-/-^ mice were extremely high, with a median of 20 ng/ml (**Figure 4D**). It is noteworthy that the increase in Cxcl13 begins around week 20, coinciding with weight loss in *Bcl2*-Tg/*Tfl* ^-/-^ mice, followed by an early death (**Figures 3C**, **4E**).

### B220^-^IgM^+^ cells are the main producer of Cxcl13.

**Figure 5:**
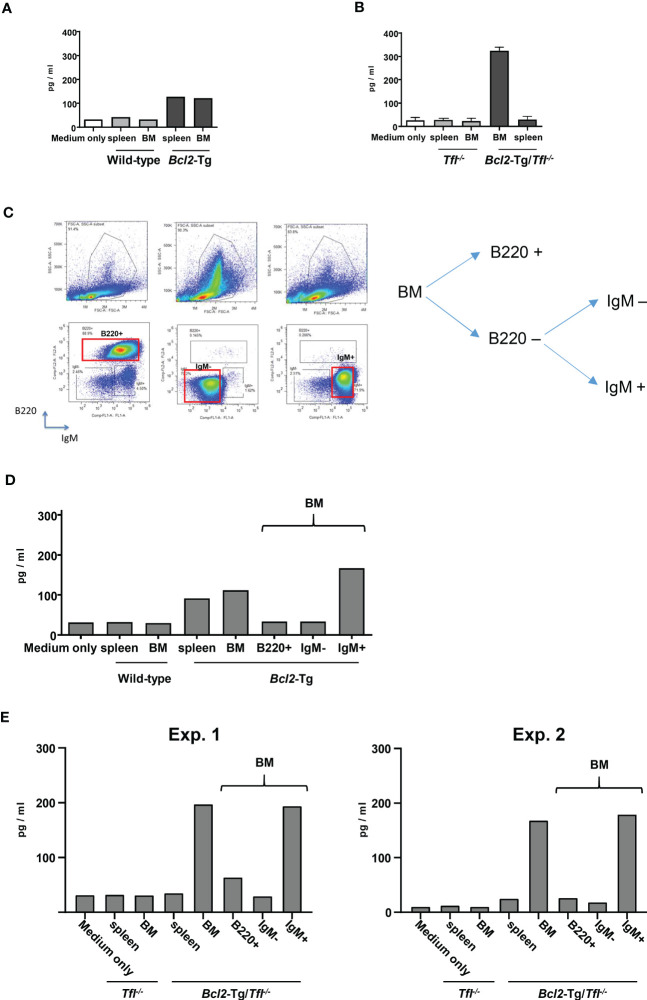
B220^-^IgM^+^cells are the main producers of Cxcl13. **(A)** ELISA assay to detect Cxcl13 of supernatant from cultured cells. Bone marrow (BM) and splenocytes from wild-type (WT) or *Bcl2*-Tg mice were cultured, and the supernatant of each cultured cell was provided for the measurement. Medium only was put as a reference. **(B)** Cxcl13 from BM and spleen cells of *Tfl* ^-/-^, or *Bcl2*-Tg/*Tfl* ^-/-^ mice (N=2). The assay was performed similarly to **(A)**. **(C)** Representative plot for B220^+^, IgM^-^, or IgM^+^ fraction. The fraction gated as a red rectangle was provided for the following analysis. **(D)** CXCL-13 measurement from the supernatant of cultured cells of wild-type (WT) and *Bcl2*-Tg mice. B220+, IgM-, or IgM+ cells were provided with cell sorting indicated as **(C)**. **(E)** Cxcl13 measurement from the supernatant of cultured cells of *Tfl* ^-/-^ or *Bcl2*-Tg/*Tfl* ^-/-^ mice. Two independent data are shown (Exp.1 and Exp. 2).

Next, we asked whether B220^-^IgM^+^ cells are the main producers of Cxcl13. We first tested the culture assay of bone marrow and solenocytes of wild-type or *Bcl2*-Tg mice to address this question. The supernatant showed a significantly increased concentration of Cxcl13 from bone marrow cells and splenocytes in the *Bcl2*-Tg mice group (**Figure 5A**). Next, we examined Cxcl13 secretion from bone marrow and splenocytes of either *Tfl^-/-^* or *Bcl2*-Tg/*Tfl^-/-^* mice. In *Bcl2*-Tg/*Tfl^-/-^* mice, the bone marrow was the main producer of Cxcl13 secretion (**Figure 5B**). Subsequently, we sorted bone marrow cells according to B220 and/or IgM expression (B220^+^, IgM^-^, or IgM^+^; **Figure 5C**) and cultured each fractionated cells to measure the Cxcl13 secretion. The supernatant of each cell population was analyzed and tended to increase Cxcl13 secretion from B220^-^IgM^+^ cells in both *Bcl2*-Tg and *Bcl2*-Tg/*Tfl^-/-^* mice with 2 independent experiments (IgM^+^; **Figures 5D, E**). Thus, B220^-^IgM^+^ cells in the bone marrow are considered to be the main producers of Cxcl13.

### TFL binds to 3’ UTR of CXCL13 mRNA to degrade in B lineage cells.

**Figure 6:**
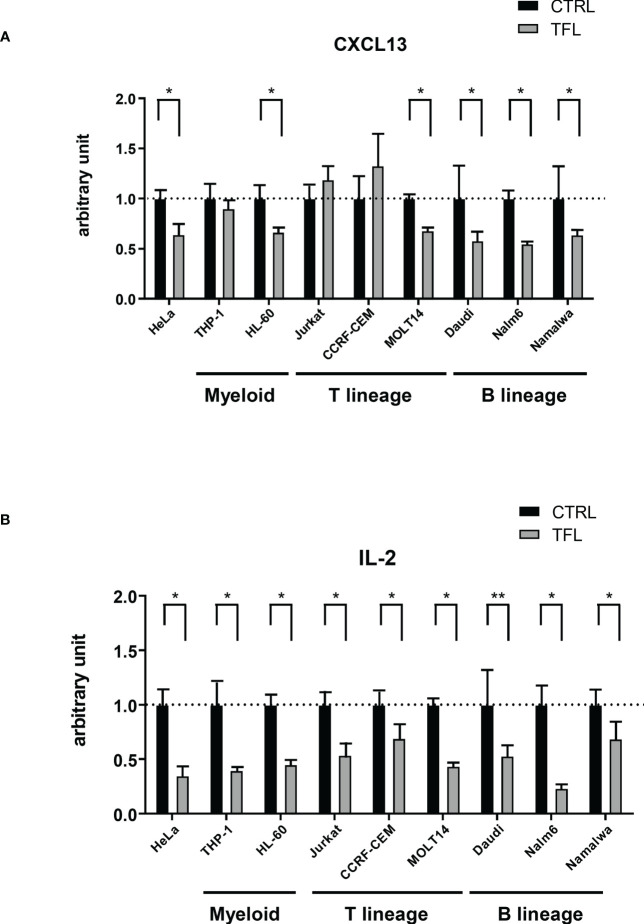
TFL degrades CXCL13 *via* 3’UTR. GFP mock (control) or GFP conjugated human TFL (TFL) vectors were transfected to several cell lines with Renilla luciferase conjugated with or without 3’UTR of CXCL13 or IL-2. Results of reporter assay of Renilla luciferase conjugated 3’UTR of CXCL13 **(A)** or IL-2 **(B)** were shown. Obtained luminescence data (Renilla luciferase/CXCL13 3’UTR and IL-2 3’UTR) were normalized by firefly luciferase as an internal control. The data were standardized by the data with the control vector, and the average of control data was arbitrarily adjusted to 1 for comparison. Experiments were repeated 3 times, and the *p*-value is shown as *<0.05 and **<0.01.

Finally, to determine whether TFL regulates CXCL13 mRNA through the degradation of mRNA 3’UTR, we performed a reporter assay with a luciferase vector containing 3’ UTR of CXCL13 mRNA. Co-transfection with the TFL expression vector showed decreased luciferase activity compared to the controls in all B lineage lines studied (Daudi, Nalm6, Molt-13, and Namawala) (**Figure 6A**), suggesting that TFL regulates CXCL13 *via* 3’UTR mRNA degradation in B lineage cells. These phenomena were not, however, seen in some other cell lines of other lineages, including THP-1, Jurkat, or CCRF-CEM. TFL did regulate IL-2 in all tested cell lines (**Figure 6B**). CXCL13 regulation seems to be restricted mainly in B cell lineage cells, while IL-2 regulation occurs promiscuously.

## Discussion

This study found that loss of *TFL* was seen in 13.6% of human mature B-cell neoplasms. While deletion of TFL does not affect lymphomagenesis in *Bcl2*-Tg FL model mice, Tfl deficiency upregulates Cxcl13 secretion significantly from the B220^-^IgM^+^ bone marrow cells and induces cachexia followed by early death. TFL loss seen in several lymphoma can be associated with an inflammatory response rather than lymphomagenesis.

TFL was identified in a transformed FL patient who has t (2;6)(p12;q23) (8, 9). It is expressed mainly in lymphoid tissue, including the thymus, spleen, and bone marrow (10). Interestingly, T or B cell activation induced its expression in cytoplasmic granules where TFL binds to 3’UTR of several cytokines, including IL-2, IL-6, TNF-α, IL-17a, c-fos, NF-κB, and IL-1β and degrades them (10, 12, 19). While TFL has been implicated in cancer prognosis, including endometrial cancer (20) and lung adenocarcinoma (21, 22), it has also been associated with autoimmune disorders through cytokine modulation. For example, *Tfl*-deficit mice prolonged encephalitic inflammation caused by the experimental animal encephalopathy model failing to degrade IL-17a in CNS-infiltrated lymphocytes (12). In addition, the hypermethylated promotor region of the *TFL* gene could be suggested in the occurrence of leukoaraiosis, neuroimaging abnormalities of the cerebral white matter in the elderly, involving aberrant inflammation-associated signaling pathways (23). A recent report showed that TFL is downregulated in GM-CSF-secreting effector memory CD4+ T-cells, which have a critical role in multiple sclerosis, controlling several cytokines, including TNFα, IL-22, and CSF2 (24). TFL is considered one of the coordinators for the expression of proinflammatory cytokines through the regulation of mRNA, tuning immune and inflammatory responses according to the integration of multiple regulatory processes in a timely and efficient manner (25). However, we have little evidence of how TFL coordinates the expression of those target genes in each cell. We found that the degree of weight loss differed among the mice (**Figure 3C**), and the degree of elevation of Cxcl13 and the timing of the onset of elevation also varied widely among individuals (**Figures 4D, E**). Indeed, some *Bcl2*-Tg/*Tfl* ^-/-^ mice can survive as *Bcl2*-Tg mice. These results suggest that there may be differential susceptibility to the loss of function of TFL. The impact of TFL function may vary between sex since many autoimmune diseases have been reported to have sex bias (26, 27). We observed female *Bcl2*-Tg/*Tfl* ^+/-^ mice died earlier than male mice indicating female is more susceptible to the effect of Tfl. We also observed that TFL regulates CXCL13 expression primarily in B lineage cell lines, while TFL degrades IL-2 in any cell lines (**Figure 6**). It is conceivable that mRNA regulators precisely control cytokines in each cell lineage to orchestrate the entire inflammatory response. We demonstrated that the loss of *TFL* was not seen in low-grade FL, and TFL expression was increased in higher-grade FL and DLBCL. But TFL expression was reduced in some DLBCL. This may suggest that TFL expression increases according to FL development, and TFL deletion occurs during the transformation from FL to DLBCL or after DLBCL development. We previously demonstrated that the loss of *TFL* facilitates cell proliferation. In this study, we observed an increased level of Ki-67 in the spleen, lymph node, and bone marrow in *Bcl2*-Tg/*Tfl* ^-/-^ mice. In addition, germinal follicles might be decreased, and NFκB activation was increased in the spleen, suggesting the transformation of follicular lymphoma. Although we did not observe an apparent morphological and histopathological difference in spleen, lymph node and bone marrow in both strains, the loss of *Tfl* might affect tumor progression in *Bcl2*-Tg/*Tfl* ^-/-^ mice. It is also of interest whether germinal center B cells and germinal center follicular helper T cells reduced in number in VavP-*Bcl2*-Tg/*Tfl* ^-/-^ spleen.

CXCL13 expression in B cell lymphoma is uncommon; however, some reports suggested the association between CXCL-13 expression and lymphoma development. Husson et al. showed that CXCL13 secretion was seen in FL cells (5). Another report demonstrated higher serum CXCL13 levels for more than 3 years increased the risk of development of HIV-associated non-Hodgkin lymphoma (6). In addition, a more recent survey including 67 immune and inflammation markers between 301 patients with non-Hodgkin lymphoma diagnosed 5+ years after blood collection showed that higher serum CXCL13 level predicts future occurrences of DLBCL (7). Therefore, it seems reasonable to suggest that several cytokines, including CXCL13, can be upregulated in non-Hodgkin lymphoma. It needs to be investigated whether the inflammatory signaling impacts lymphoma prognosis in the future.

CXCL13 in cerebrospinal fluid is a prognostic marker in Clinically Isolated Syndrome regarding conversion to multiple sclerosis (28). Also, high expression of CXCL13 worsens the prognosis of gastric cancer after resection (29). Since higher CXCL13 expression also worsens rheumatoid arthritis or multiple sclerosis, its function is considered to promote inflammatory pathways. Our serum Cxcl13 expression level was extremely high (median concentration>20 ng/ml, **Figure 4D**). Even Cxcl13 transgenic mice in the thymus demonstrate a maximum serum concentration of <20 ng/ml during 30 to 40 weeks (30). Therefore, it is plausible that *Tfl* deficiency induced extraordinary Cxcl13 secretion in B220^-^IgM^+^ cells in the bone marrow, which possibly causes weight loss, so-called cachexia, and worsens survival. These indicate some inflammatory deterioration rather than tumor progression could worsen the survival of the mice. In fact, we saw the reduced platelet counts, MCV, and MCH in the peripheral blood of *Bcl2*-Tg/*Tfl^-/-^* mice at 35-45 weeks but not at 20-30 weeks, suggesting *Bcl2*-Tg/*Tfl^-/-^* mice suffered from more severe inflammation than *Bcl2*-Tg mice during this period. This may be an example of how mRNA regulators affect the cancer environment through inflammatory processes caused by cytokine dysregulation.

*Bcl2*-Tg mice are known to develop FL with prolonged germinal center reactions. *Vav* gene promoter enabled Bcl-2 expression mainly in hematopoietic cells but not in non-hematopoietic tissue (31). While most of these animals developed FL after 40 weeks, we found a unique cell population in their bone marrow, which expressed IgM but not B220 (IgM^+^B220^-^). We found a similar population in the spleen, thymus, and lymph nodes, although the cell population in the bone marrow was more than that in any other organs. Based on phenotypic findings of the bone marrow cells, IgM^+^B220^-^ population seems to be monocyte/macrophage lineage cells. CD25 has been reported to express on monocyte (32) and under certain conditions, such as tumor environment. Immunoglobulin can also be expressed in monocyte (33). This population partially expresses Gr-1, which recognizes Ly6G and Ly6C. This could underscore the monocyte/macrophage phenotype since Ly6C is a marker for monocyte/macrophage. On the other hand, this B220^-^IgM^+^ cell may represent antibody-secreting cells (ASCs), including plasmablast and plasma cells, since lower B220 expression has been reported in CD138^high^IgM^high^ ASCs (34). In addition, B220 expression is further diminished in bacterial-infected mice. Our cDNA array data showed ASC-related genes such as Prdm1, Sdc1, and Ell2 (35) were slightly upregulated in *Bcl2*-Tg/*Tfl^-/-^* mice compared with *Bcl2*-Tg mice (x1.1-x4.7, data not shown) suggesting the skew to ASC in this population. However, our data shows CD138 expressed in a small distinctive population in the bone marrow of *Bcl2*-Tg mice (3.2%, data not shown). Therefore, it is less likely that the main population is ASCs. Interestingly, in our report, these abnormal cells were the main producer of Cxcl13. Thus, disease progression (early death) could be caused by metabolic factors of the cancer-bearing individuals. The abnormal B cell population may induce loss of Tfl to promote its own proliferation, but the induced inflammation may be too strong to cause individual death. Further studies are necessary to confirm if Cxcl13 blockade (36) in this model may prolong survival by alleviating the excessive inflammatory processes.

In summary, although precise mechanisms of function are still unknown, the TFL-CXCL13 axis could be an example of how inflammatory processes affect the outcome of lymphoma patients. Our findings provide insight into cytokine regulation *via* mRNA degradation in a mouse lymphoma model.

## Data availability statement

The original contributions presented in the study are included in the article/**Supplementary Materials**, further inquiries can be directed to the corresponding author/s.

## Ethics statement

The studies involving human participants were reviewed and approved by Kobe University Hospital. The patients/participants provided their written informed consent to participate in this study. The animal study was reviewed and approved by Kobe University Hospital.

## Author contributions

Contribution: KM and KW performed all experiments and wrote the manuscript. CF, YuK, HK, TS, SI, AS, SN, and NA helped with animal maintenance, tissue sample preparation, and capturing the images. and YoK supervised the studies for *Bcl2*-Tg and *TFL^-/-^* mice. and TM supervised all experiments and wrote the manuscript. All authors contributed to the article and approved the submitted version.
