# Supplementary material for: Tfl deletion induces extraordinary Cxcl13 secretion and cachexia in VavP-Bcl2 transgenic mice
Source: Front Immunol. 2023 May 26;14:1197112. doi: 10.3389/fimmu.2023.1197112 (PMC10250710; doi:10.3389/fimmu.2023.1197112)

Supplemental Fig 1

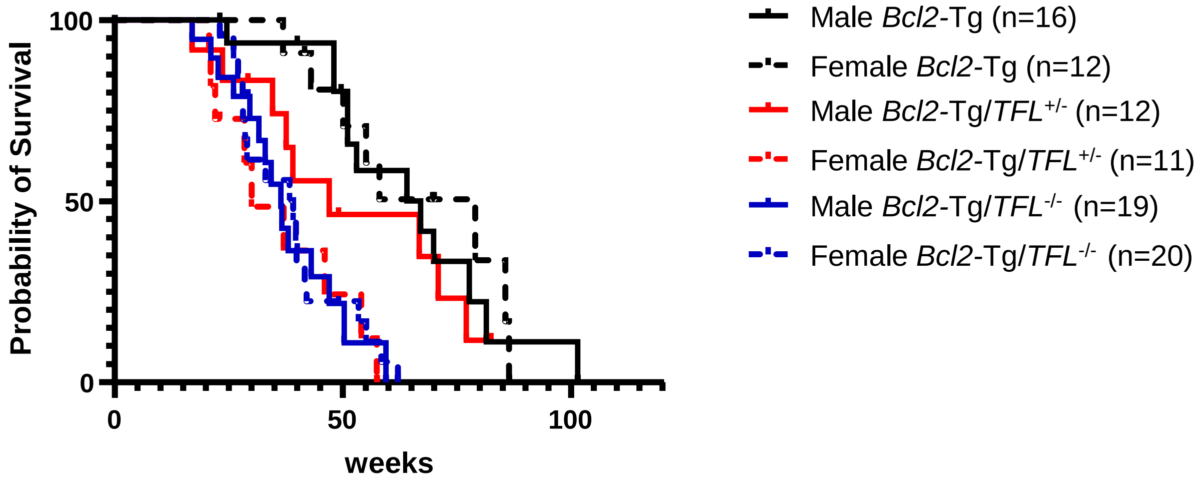

Male *Bcl2*-Tg vs. Female *Bcl2*-Tg : ns
Male *Bcl2*-Tg/*TFL*$^{+/-}$ vs. Female *Bcl2*-Tg/*TFL*$^{+/-}$:$p<0.05$
Male *Bcl2*-Tg/*TFL*$^{-/-}$ vs. Female *Bcl2*-Tg/*TFL*$^{-/-}$: ns

Supplemental Fig 2

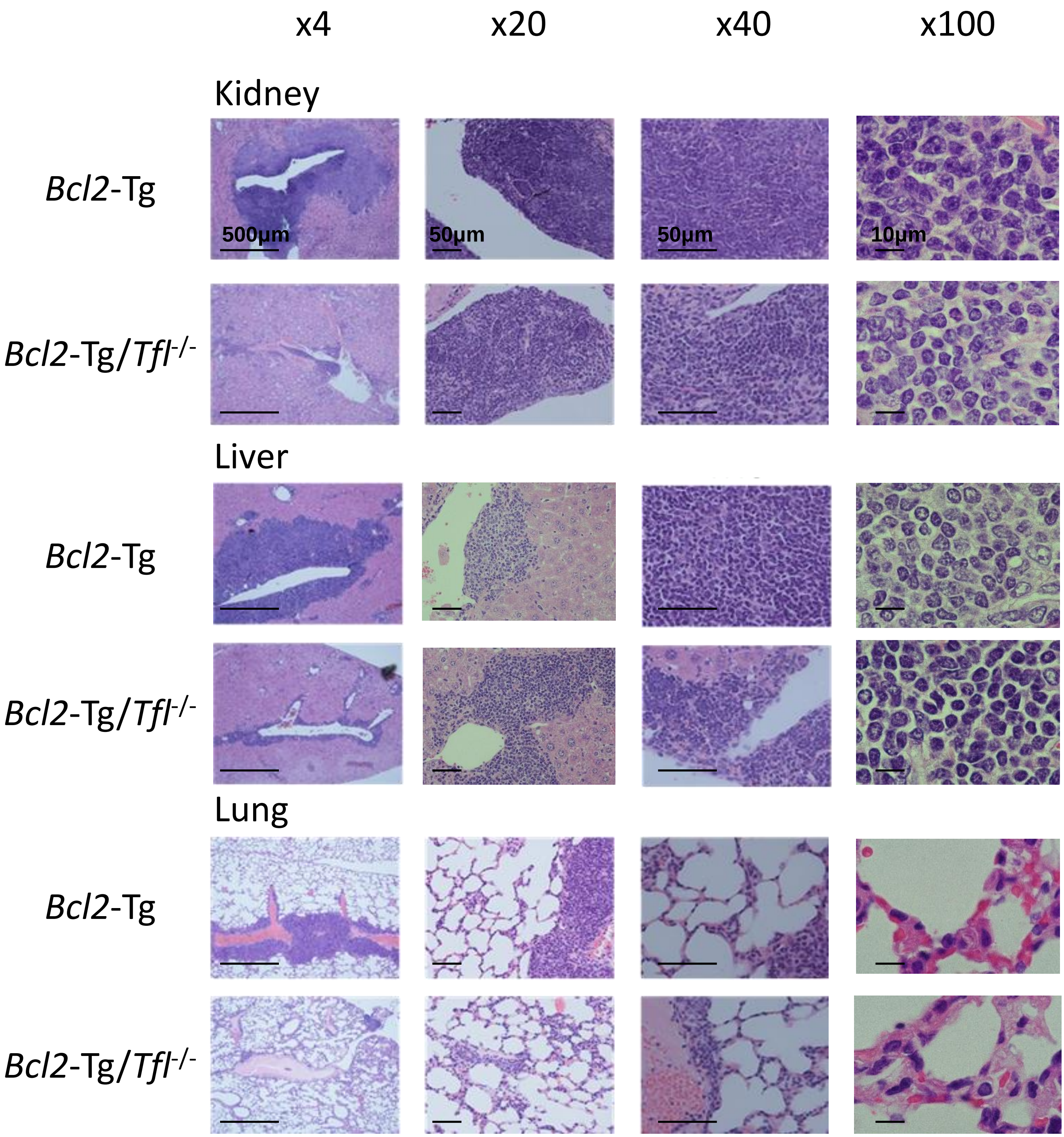

Supplemental Fig 3

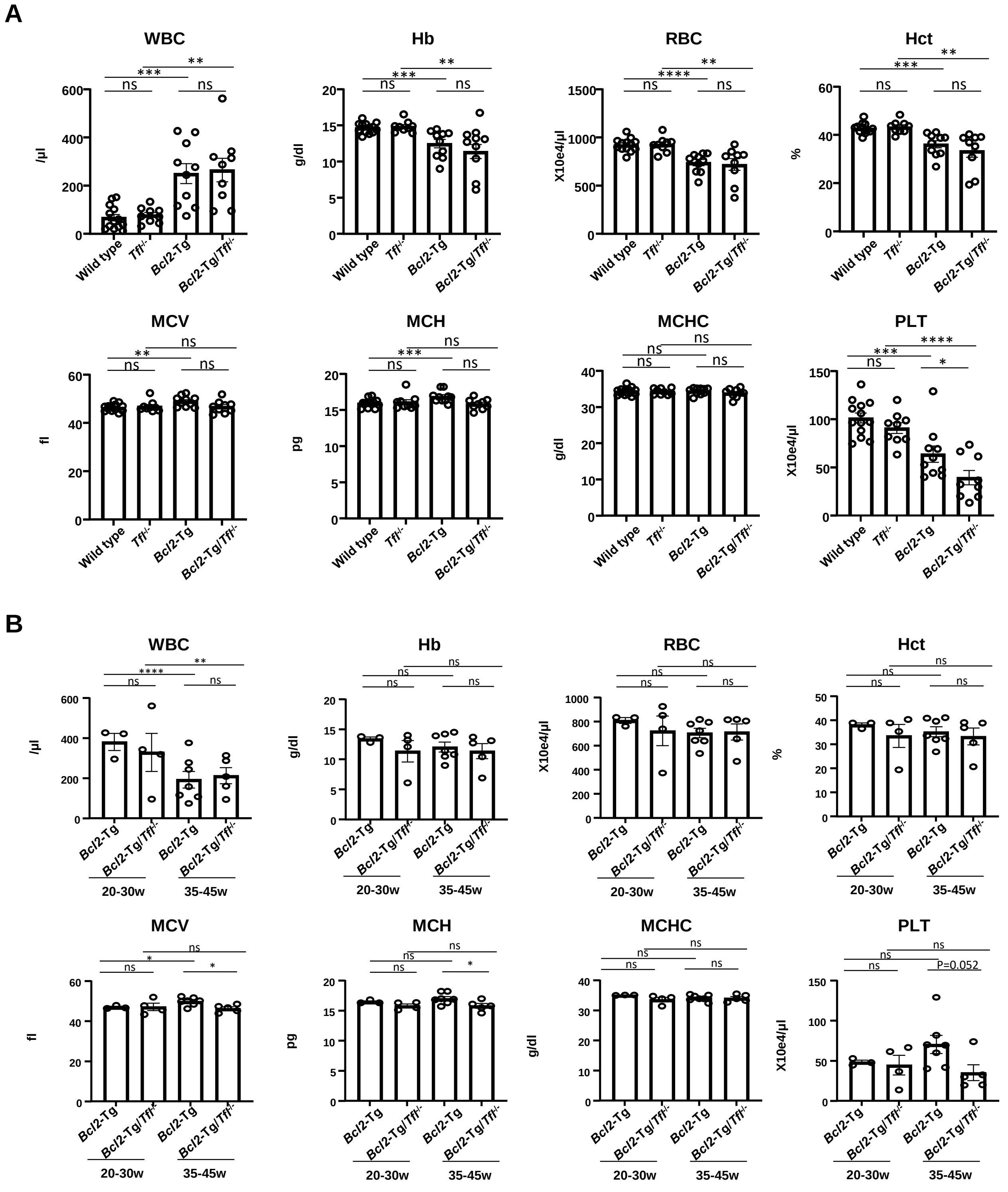

Supplemental Fig 4

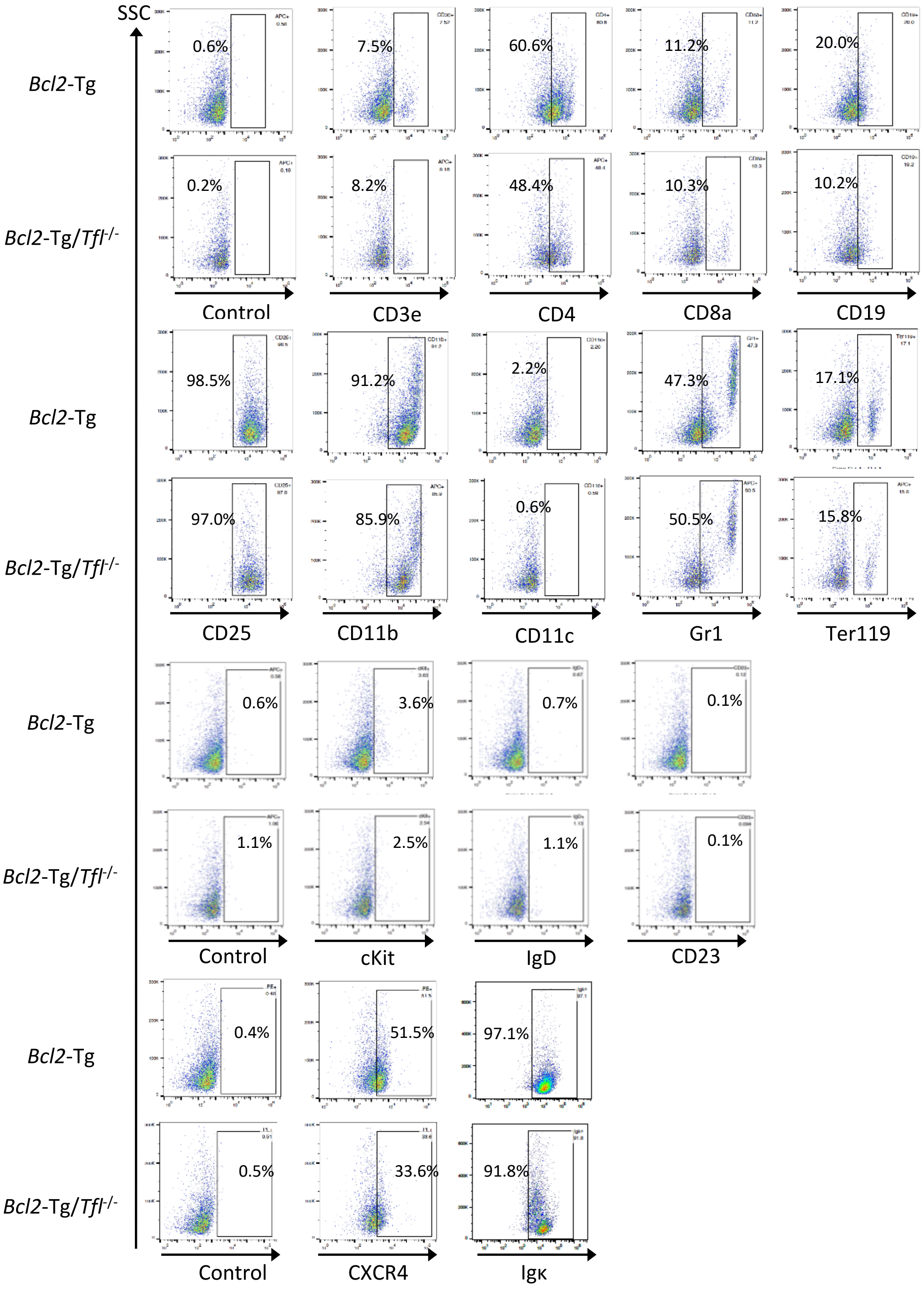

Supplement: Supplementary file 1 [file DataSheet_1.pdf]
