# Supplementary material for: Tfl deletion induces extraordinary Cxcl13 secretion and cachexia in VavP-Bcl2 transgenic mice
Source: Front Immunol. 2023 May 26;14:1197112. doi: 10.3389/fimmu.2023.1197112 (PMC10250710; doi:10.3389/fimmu.2023.1197112)
Supplement: Supplementary file 2 [file Table_1.docx]

# **Supplemental Information**

***TFL* deletion induces extraordinary Cxcl13 secretion and cachexia in VavP-*Bcl2* transgenic mice**

Kentaro Minagawa^1,2^, Kanako Wakahashi^1^, Chie Fukui^1^, Yuko Kawano^1^, Hiroki Kawano^1^, Tomohide Suzuki^1^, Shinichi Ishii^1^, Akiko Sada^1^, Shinichiro Nishikawa^1^, Noboru Asada^1^, Yoshio Katayama^1^, Toshimitsu Matsui^3^

**Affiliations:**

1. Hematology, Department of Medicine, Kobe University Graduate School of Medicine, 7-5-1 Kusunoki-cho, Chuo-ku, Kobe 650-0017, Japan
2. Hematology & Oncology Division, Penn State College of Medicine, 500 University Drive, Hershey, PA 17033, USA
3. Department of Hematology, Nishiwaki Municipal Hospital, 652-1 Shimotoda, Nishiwaki 677-0043, Japan

Supplemental information includes two tables, one file, and four figures.

*Correspondence: Kentaro Minagawa, MD, PhD

Hematology & Oncology Division, Penn State College of Medicine, 500 University Drive, Hershey, PA 17033, USA

Tel: +1-717-531-0003

Email: kminagawa@pennstatehealth.psu.edu

# **Supplemental Table 1. Results of *TFL* deletion in bone marrow samples.**

Disease characteristics in BM samples

| Disease | N | **CEP=LSI** | **CEP>LSI** | **CEP<LSI** |
| --- | --- | --- | --- | --- |
| Acute lymphoblastic leukemia | 19 | 18 | 1 | 0 |
| Chronic lymphocytic leukemia | 7 | 5 | 2 | 0 |
| B cell lymphoma* | 7 | 5 | 2 | 0 |
| Diffuse large B cell lymphoma | 29 | 27 | 1 | 1 |
| Follicular lymphoma | 18 | 16 | 2 | 0 |
| MALT lymphoma | 3 | 3 | 0 | 0 |
| Mantle cell lymphoma | 4 | 3 | 1 | 0 |
| Lymphoplasmacytic lymphoma | 5 | 3 | 1 | 1 |
| Monoclonal gammopathy unknown significance | 5 | 5 | 0 | 0 |
| Multiple myeloma | 46 | 39 | 7 | 0 |
| NK/T cell lymphoma | 3 | 3 | 0 | 0 |
| Angioimmunoblastic T cell lymphoma | 1 | 1 | 0 | 0 |
| Adult T cell leukemia/lymphoma | 4 | 4 | 0 | 0 |
| Peripheral T cell lymphoma | 7 | 7 | 0 | 0 |
| Hodgkin lymphoma | 6 | 6 | 0 | 0 |
| Total | 164 | 145 | 17 | 2 |

*subtype is not determined

# **Supplemental Table 2. Results of TFL deletion in lymph nodes or tissue samples.**

Disease characteristics in lymph nodes or tissue samples

| Disease | N | **CEP=LSI** | **CEP>LSI** | **CEP<LSI** |
| --- | --- | --- | --- | --- |
| Malignant lymphoma* | 5 | 4 | 1 | 0 |
| Chronic lymphocytic leukemia | 3 | 3 | 0 | 0 |
| B-cell lymphoma* | 1 | 1 | 0 | 0 |
| Burkitt lymphoma | 1 | 0 | 0 | 1 |
| Diffuse large B-cell lymphoma | 40 | 33 | 5 | 2 |
| Follicular lymphoma | 31 | 25 | 6 | 0 |
| Mediastinal B-cell lymphoma | 1 | 1 | 0 | 0 |
| MALT lymphoma | 6 | 6 | 0 | 0 |
| Mantle cell lymphoma | 3 | 2 | 1 | 0 |
| Lymphoplasmacytic lymphoma | 1 | 1 | 0 | 0 |
| Multiple myeloma | 1 | 1 | 0 | 0 |
| NK/T cell lymphoma | 2 | 1 | 1 | 0 |
| Anaplastic large cell lymphoma | 1 | 1 | 0 | 0 |
| Angioimmunoblastic T-cell lymphoma | 4 | 4 | 0 | 0 |
| Adult T-cell leukemia/lymphoma | 2 | 2 | 0 | 0 |
| Peripheral T-cell lymphoma | 6 | 4 | 2 | 0 |
| Hodgkin lymphoma | 15 | 12 | 2 | 1 |
| Total | 123 | 101 | 17 | 4 |

*subtype is not determined

**Supplemental file 1**

The complete list of cDNA arrays in comparison between *Bcl2*-Tg and *Bcl2*-Tg/*Tfl*^-/-^ (experiment 1)

**Supplemental file 2**

The complete list of cDNA arrays in comparison between *Bcl2*-Tg and *Bcl2*-Tg/*Tfl*^-/-^ (experiment 2)

**Supplemental file 3**

The list of 182 genes in *Bcl2*-Tg/*Tfl*^-/-^ mice that were upregulated at least twice than *Bcl2*-Tg mice

**Supplemental figure legend**

**Supplemental Fig 1. Survival curve comparison of *Bcl2*-Tg, *Bcl2*-Tg/*Tfl*^+/-^, or *Bcl2*-Tg/*Tfl*^-/-^ male and female mice**

Survival curve is shown for male *Bcl2*-Tg (N=16), *Bcl2*-Tg/*TFL*^+/-^ (N=12), or *Bcl2*-Tg/*TFL*^-/-^ (N=19) mice and female *Bcl2*-Tg (N=12), *Bcl2*-Tg/*TFL*^+/-^ (N=11), or *Bcl2*-Tg/*TFL*^-/-^ (N=20) mice. The *p*-value for comparison is shown in the column.

**Supplemental Fig 2. Hematoxylin and Eosin (HE) staining of *Bcl2*-Tg, or *Bcl2*-Tg/*TFL*^-/-^ mice**

HE staining for kidney (A), liver (B), or lung (C) is shown.

**Supplemental Fig 3. Peripheral blood counts of *Bcl2*-Tg/*Tfl*^+/+^ and Bcl2-Tg/*Tfl*^-/-^ mice**

Complete blood counts (CBC) in wild-type (n=14), *TFL*^-/-^(N=9), *Bcl2*-Tg (N=10), and *Bcl2*-Tg/*Tfl*^-/-^ (N=9) mice are shown in (A). Comparing CBC at 20-30 weeks (20-30w) and 35-45 weeks (35-45w) of *Bcl2*-Tg and *Bcl2*-Tg/*Tfl*^-/-^ mice are shown in (B). The *p*-value is shown as *<0.05, **<0.01, ***<0.001, and ****<0.0001. WBC: White blood cell, Hb: Hemoglobin, RBC: Red blood cell, Hct: Hematocrit, MCV: Mean Corpuscular Volume, MCH: Mean Corpuscular Hemoglobin, MCHC: Mean Corpuscular Hemoglobin Concentration, PLT: Platelet

**Supplemental Fig 4. Phenotypic analyses of** ***Bcl2*-Tg/*Tfl*^+/+^ and Bcl2-Tg/*Tfl*^-/-^ mice**

Flow cytometry analysis of the B220^-^IgM^+^ fraction in the bone marrow of *Bcl2*-Tg/*Tfl*^+/+^ and Bcl2-Tg/*Tfl*^-/-^ mice are shown in the figure. Representative plots were shown.
